# Nutritional, Antibacterial and Thrombolytic Prospects of Freshwater *Paludomas conica* Protein Hydrolysate and Its Anti‐Inflammatory Potential in LPS‐Induced RAW 264.7 Macrophage Cells

**DOI:** 10.1002/fsn3.4711

**Published:** 2024-12-31

**Authors:** Tanvir Ahmed Siddique, Khalid Juhani Rafi, Sumaiya Akter, Farhana Yesmin Bithy, Rasheda Aktar, Asif Nadim Khan, Mumtahina Majid, Farjana Sultana, Srabonti Saha, A. M. Abu Ahmed, Atiar Rahman

**Affiliations:** ^1^ Department of Biochemistry and Molecular Biology University of Chittagong Chittagong Bangladesh; ^2^ Department of Genetic Engineering and Biotechnology University of Chittagong Chittagong Bangladesh

**Keywords:** antihemolytic, anti‐inflammation, LPS‐stimulated RAW264.7 macrophage, nutritional value of *Paludomas conica*, Protamex, protein hydrolysate

## Abstract

Chronic inflammation and heme‐iron overload can result from bacterial hemolysis. Along with the synthetic drugs, numerous traditional and functional food approaches are equally trialed to eradicate the problem. As a prospective new source of dietary protein hydrolysates, freshwater mollusks (*Paludomas conica*) have recently drawn huge interest from researchers. In this research, protein hydrolysate (PhPC) of *Paludomas conica*, prepared by the enzyme digestion method, was analyzed for proximate nutritional and minerals contents and deciphered its suppressive effects on inflammatory gene expression in LPS‐stimulated RAW264.7 macrophage cells. The inhibitory action of protein denaturation is also unfolded with established in vitro and in vivo models. Anti‐hemolytic, antibacterial, and thrombolytic effects of PhPC were respectively assessed by H_2_O_2_‐induced hemolysis of RBCs, the disc diffusion method, and the clot lysis method. The proximate nutritional and mineral contents of PhPC revealed it to be an enriched source of nutrients, crude protein, carbohydrates, Calcium, and Magnesium. Heavy metals were found to be within the prescribed limit. The PhPC suppressed the expression of inflammatory genes, including *COX‐2*, *iNOS*, *IL‐6*, *TNF*‐α, and IL‐1, multifold in LPS‐stimulated RAW264.7 macrophages. The inhibition concentrations (IC_50_) of PhPC in the bovine serum albumin denaturation inhibition test and membrane stabilization tests were 431.39 and 285.25 μg/mL, respectively. The PhPC was discerned to be active against *Shigella flexneri, Pseudomonas aeruginosa,* and *Shigella dysenteriae* ; its maximum thrombolytic effect was displayed to be 23.72% ± 2.71%. The findings demonstrate that the nutritionally enriched PhPC could be affirmed as an exciting invertebrate anti‐inflammatory agent Extending other biological functions needs to be further characterized with its pure protein or protein products.

## Graphical abstract

Nutritional and biological potential of a freshwater mollusk, *Paludomas conica*, is revealed in this research. Protein hydrolysate of the mollusk has been used as an experimental sample. Proximate nutritional, mineral, and heavy metal contents, as well as prevalently anti‐inflammatory effects in LPS‐induced RAW 264.7 macrophage cells, were enumerated in this research along with some other biological effects.

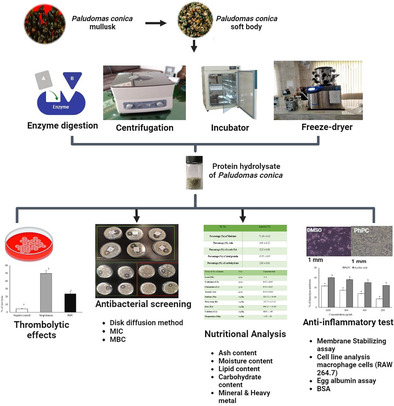

**Abbreviations**
°C: degree celscius
DMSO: dimethyl sulfoxide
Gram (−): gram negative
Gram (+): gram positive
IC50: inhibition concentration 50
LC50: lethal concentration 50
M: molar
mg/kg: milligram per kilogram
min: minute
mL: milliliter
mM: milli molar
NaCl: sodium chloride
NaOH: sodium hydroxide
Nm: nanometer
pH: negative logarithm of hydrogen ion concentration
PhPC: *Paludomas conica* protein hydrolysates
ppm: parts per million
UV: ultraviolet
v/v: volume by volume
w/v: weight by volume
w/w: weight by weight
μg: microgram
μg/mL: microgram per Milliliter
μL: microliter

## 1 Introduction

For nearly a century, there has been evidence linking bacterial co‐infection to infection‐related hemolysis (Dimitrov et al. 2023). Several bacterial infections are reported to cause pathological hemolysis, which eventually creates heme‐iron overload leading to the production of inflammation (Zhong et al. 2018). Inflammation, which is an immunological reaction triggered by noxious stimuli, serves to restore tissue homeostasis when there is an infection or injury. Since the macrophages and other specialized phagocytic cells remove defective RBCs from the circulation, the anti‐inflammatory defense strategy entails changes to vascular permeability, the attraction and concentration of immune cells, and the release of pro‐inflammatory mediators at immune reaction sites. Among inflammation‐related processes, macrophages play a significant role in inflammatory disorders through the secretion of reactive oxygen species (ROS) (Mittal et al. 2014). These cells also produce other inflammatory mediators, including prostaglandins and reactive nitrogen species (RNS), such as nitric oxide (NO) (Lugrin et al. 2014). Stimulated macrophage cells release cytokines such as interleukin 6 (IL‐6), interleukin 10 (IL‐10), 1β (IL‐1β), and tumor necrosis factor α (TNF‐α), which are involved in the regulation of inflammation (Arango Duque and Descoteaux 2014; Lawrence and Fong 2010). Despite a lot of commercially available anti‐inflammatory drugs, almost all of them are associated with adverse side effects due to their prolonged consumption. Therefore, natural biometabolites exhibiting anti‐inflammatory activity as functional foods or nutraceutical formulations exponentially emerged due to their safer modes and variety of immunomodulatory properties.

Along with the plant bioactive anti‐inflammatory agents, invertebrates have been greatly attracted and focused on, while marine mollusks orchestrate a novel strategy for therapeutic applications (Elbandy 2022). Their protein sources, especially the protein hydrolysates, a complex mixture of peptides with different sizes and compositions, as well as other free amino acids, enzymes, and nonreacted native proteins, are rich sources of bioactive peptides, which have been shown to be anti‐thrombotic, anti‐hypertensive, anti‐microbial, anti‐cancer, anti‐oxidative, and immunomodulatory (Leal et al. 2012; Sánchez and Vázquez 2017; Senthilkumar and Kim 2013). Mollusks are the most important resource of all types of seafood in Southeast Asian countries. Marine and freshwater mollusks have provided alkaloids, terpenes, steroids, aliphatic hydrocarbons, carbohydrates, amino acids, peptides, and proteins (Calado et al. 2022; Senthilkumar and Kim 2013). Some of the marine mollusks (e.g., *Dicathais orbita*) act as anti‐inflammatory agents by suppressing nitric oxide (NO) and tumor necrosis factor α (TNFα) in lipopolysaccharide (LPS)‐stimulated RAW264.7 macrophages (Ahmad et al. 2017). However, very few of freshwater snails (*Pila globose, Lissachatina fulica*) have so far been studied for their anti‐inflammatory as well as other biological effects (Srinandhinidevi and Viveka 2021; Wiya, Nantarat, and Saenphet 2020). And the comprehensive chemical investigation for the vast array of their bioactive and therapeutically lead metabolites is still insufficiently evident (Ciavatta et al. 2016; Khan and Liu 2019). The protein hydrolysates from *Paludomas conica*, a novel invertebrate source, have thus been presented as a therapeutically important new source in this research.

*Paludomas conica* is one of the most prevalent snail species in South Asia. Indigenously it is known as Paba Shamuk which falls under the *Paludomas* genus and Paludomidae family of tropical freshwater gastropod mollusks. In the Hill Tracts region of Bangladesh, *Paludomas* are regularly consumed by the tribe people for their nutritional and medicinal value and have faith in nature's therapeutic rules (Rafi et al. 2023). However, their biological and nutritional potentials are hardly investigated. This research studied the protein hydrolysate of *Paludomas conica* for its anti‐inflammatory effects, evaluating the mRNA expression of inflammatory genes, as well as other biological and nutritional potentials.

## 2 Materials and Methods

### 2.1 Chemicals and Reagents

In this work, analytical grade chemicals and reagents were employed unless otherwise noted. Sodium phosphate (Na_3_PO_4_), bovine serum albumin (BSA), sodium nitroprusside *N*‐(1‐naphthyl) ethylenediamine dihydrochloride, 10,000 I.U. lyophilized streptokinase, ciprofloxacin (BBL Sensi‐Disc) and Protamex were procured from Sigma‐Aldrich (Sigma‐Aldrich Inc. PO Box 14,508, St. Louis, MO 68178, USA).

### 2.2 Collection of and Identification of *Paludomas conica*

*Paludomas conica*, the snail species, was collected from Kaptai Lake, Rangamati (22°29′45″ N, 92°13′45″ E), Chittagong division, Bangladesh, in January 2020. The sample was identified and authenticated by Professor Dr. Sheikh Aftab Uddin, Director, Institute of Marine Sciences and Fisheries, University of Chittagong, Bangladesh. A specimen of the sample has been preserved in the institutional museum with the Voucher no. CUMS‐R2020/01.

### 2.3 Preparation of *Paludomas conica* Snail Protein Hydrolysate

**FIGURE 1:**
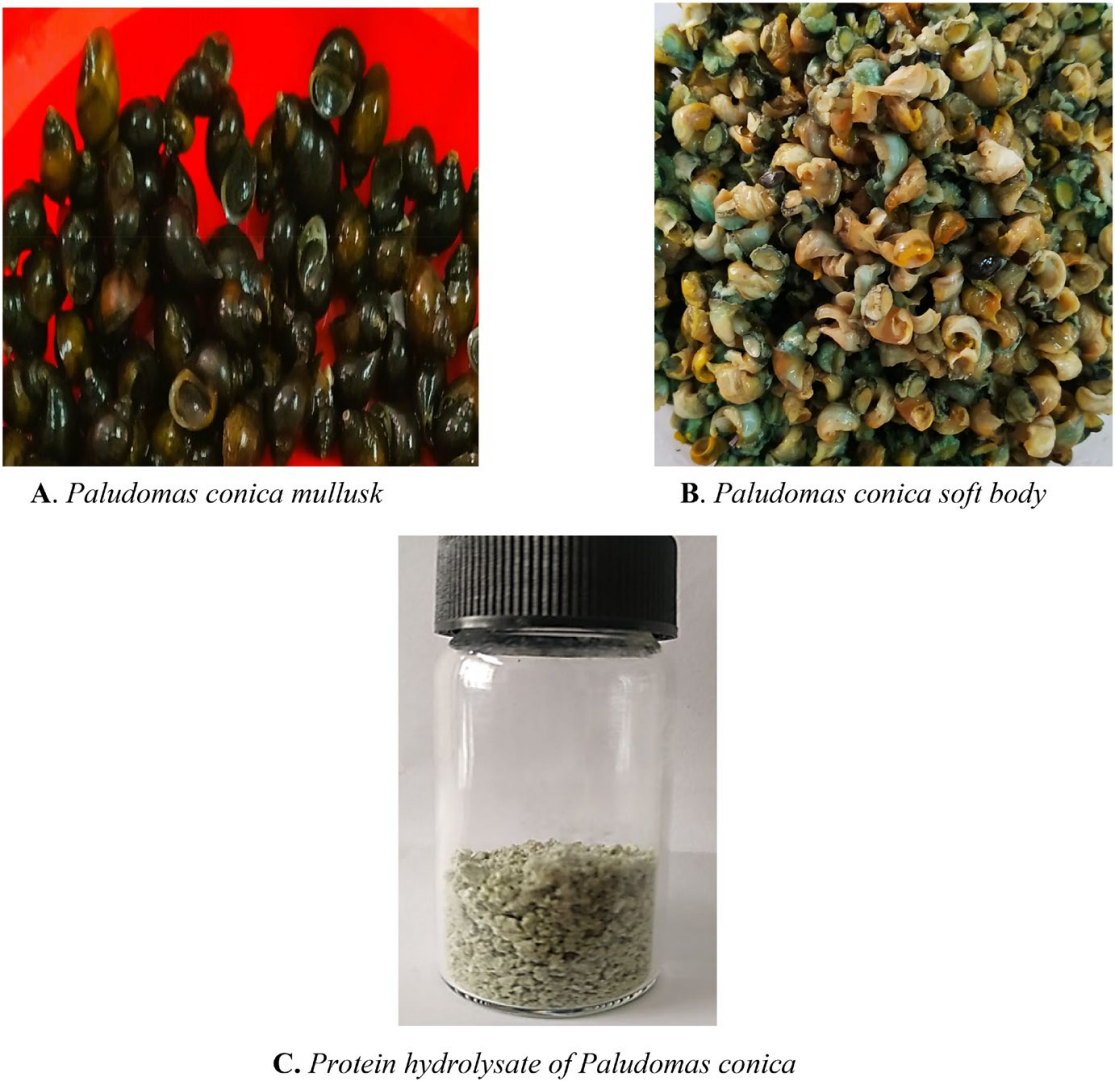
Sample specimen of *Paludomas conica* mollusk collected from fresh water of hilly canal. Figure shows (A) Intact *Paludomas conica*; (B) Soft body of *Paludomas conica* after separating the shell; (C) The protein hydrolysate after treating the homogenate of soft body using the enzyme Protamex.

The collection of *Paludomas conica* flesh and preparation of *P. conica* protein hydrolysate (PhPC) are accomplished by the methods described by Rafi et al. (2023). Briefly, shells of the snails were taken off, and the snails' flesh was carefully taken out. The flesh was washed with tap water twice or three times to get rid of sand and other dirt. The extra water was taken out, and the flesh (substrate) was stored at −20°C in polyvinyl chloride bags until it was needed (PVC). The flesh was allowed to warm up at room temperature. Then, 100 g of the flesh sample was boiled in water at 80°C for 1 h 30 min. After the sample was taken out of the water, it was put in 4.5 mL (w/v) of 0.5 M Na_3_PO_4_ buffer. By mixing 0.5 g of Protamex (Sigma‐Aldrich, MDL number: MFCD00132092, Protease from *Bacillus* sp.) with the buffer of the sample, enzyme digestion was accomplished. The ratio of substrate to the enzyme was 1:200 (w/w). Total digestion was done by putting the mixture in an incubator at 55°C for 2 h and 30 min, shaking it at 170 rpm. The enzyme‐substrate reaction was stopped by heating the mixture to 90°C for 15 min. The digested slurry mixture was centrifuged for 15 min at 2000 × *g* and 4°C. The supernatant was collected and cleaned with an MWCO ≥ 25 kDa SpectraPor dialysis membrane. It was then kept at −80°C before being freeze‐dried. The filtered supernatant was turned into a lyophilized powder using a freeze dryer (Labconco, FreeZone 4.5 Liter −50°C Benchtop Freeze Dryer, Kansas, MO, USA). The light‐green powdered *P. conica* protein hydrolysate (PhPC) was preserved at 4°C for future use. The sample specimen, flesh, and protein hydrolysate are shown in Figure 1.

### 2.4 Estimation of Protein Hydrolysate Concentration and Degree of Hydrolysis

After enzymatic digestion, the protein hydrolysate content of *P. conica* was measured using Bradford's method, using bovine serum albumin (BSA) as a reference agent (Bradford 1976).

The degree of hydrolysis (DH) was calculated by the previously described method (Tang et al. 2009). It was then heated to ideal conditions for protamex enzymes (Protamex at pH = 5.5–7.5 at temperature 35°C–60°C). From the first to the sixth hour, a continuous log of the hydrolytic yield was kept. The enzymes were inactivated after hydrolysis by bringing the pH back into equilibrium and heating the reaction liquid to 100°C for 10 min. In this study, the supernatant was obtained by centrifuging the protein hydrolysates at 10,000 × *g* for 15 min. The degree of degradation was determined using the pH‐stat method of Adler‐Nissen (1976). When the amino groups of the amino acids were combined with Sanger's reagent (1‐fluoro‐2,4‐dinitrobenzene), a yellow complex of amino acids formed, which was used to measure the DH of hydrolyzed protein (Goodwin 1968). The absorbance was measured at 410 nm.

$$\text{Degree of hydrolysis}\;\left(\%\right)=\frac{h}{h_{\text{total}}}\times 100$$

### 2.5 Nutrition Analysis

#### 2.5.1 Proximate Composition Analysis of PhPC

The methods of the Association of Official Analytical Chemists (1990) were used for the determination of moisture, protein, fat, ash, and carbohydrate. The analysis of minerals of our sample *P. conica* was determined at the Chemistry Division, Atomic Energy Centre, Dhaka (AECD), in collaboration with the Department of Biochemistry and Molecular Biology, University of Chittagong.

#### 2.5.2 Measurement of Ash Content of PhPC

The material is burned for a longer period at a high temperature to turn it into ash. The percentage of the dry sample is used to express the ash content. After coming to a boil, the porcelain crucibles used for the analysis were cleaned with distilled and demineralized water, respectively, and diluted hydrochloric acid. After that, it was dried in an oven at 120°C and burned for 3 h at 550°C in a furnace. Following their removal from the furnace, the crucibles were allowed to cool in desiccators. M1 was the measured mass of the crucibles. The mass of the samples, measured at 2 g, was entered into the porcelain crucible and designated as M2. The samples were then heated to 550°C in a furnace until the carbon was removed, and after 8 h, the residue turned grayish white. Before weighing, the samples were taken out of the furnace, put in desiccators, and given 30 min for the crucible to cool. Finally, the mass was weighed as M3. And the total ash contents of samples were calculated with the following formula:

$$\mathrm{Ash}\;\left(\%\right)=\left[\left(\text{M3}-\text{M1}\right)÷\left(\text{M2}-\text{M1}\right)\right]\times 100$$

$$\text{M1}=\text{the}\;\mathrm{dry}\;{\text{crucible}}^{'}s\;\text{mass}$$

M2=mass of the sample and the crucible

M3=mass ofashand the crucible

#### 2.5.3 Determination of Moisture Content

High temperatures cause the water to evaporate, and the percentage of moisture in the sample is used to represent this loss of water content. Five grams of the materials were precisely weighed and placed into a dry crucible. After the sample was well combined, it was dried for 6 h at 100°C. After being dehydrated for 30 min, the dried samples were cooled and then weighed again. Until a steady weight was achieved, the procedure was repeated. As a proportion of the initial sample moisture content, the weight change was computed.

The following formula is used to get the sample's moisture content:

%*W* = [(*A* − *B*)/*A*] × 100; where %*W* = percentage of the sample's moisture content, *A* = Fresh sample weight in grams, and *B* = The dried sample's weight (g).

#### 2.5.4 Estimation of the Lipid Content of PhPC

The solubility of lipids in non‐polar organic solvents, such as hexanes, petroleum ether, or supercritical liquid carbon dioxide with or without a solvent modifier, is the basis for the conventional method for determining total crude fat. After being cleaned, the extraction flasks were dried for 30 min at 105°C in a drying oven and then chilled in desiccators. Using an analytical balance, the masses of the cooled round‐bottom flasks were determined and recorded as M1. The powdered sample was precisely weighed at 3 g each thimble, with cotton lining the bottom. The Soxhlet extraction device was filled with the thimble containing the sample. After adding 150 mL of hexane solvent to each flask and letting it extract for roughly 6 h, the flask and its contents were taken out of the Soxhlet and dried for 30 min at 105°C. After that, the flasks and their contents were put in desiccators for 30 min. Each flask's mass and fat content were measured and expressed as M2. The formula was used to calculate the crude fat contents of each sample:

% of lipid content: [(M2 − M1) ÷ M] × 100; where M2 = mass of flask and lipid extract.

M1 = the dry flask's mass; *M* = Sample weight measured on a dry basis.

#### 2.5.5 Determination of Protein Content of PhPC

The protein was estimated using the Lowry technique (Lowry et al. 1951). By using his approach, the color created by the protein's biuret reaction with alkaline cupric tartrate is combined with the reduction of phosphomolybdic‐phosphotungstic components in the Folin–Ciocalteu reagent, which results in a blue result. Scooping out 100 mg of sample powder, 10 mL of 0.2 M phosphate buffer was added, and the mixture was well crushed with a mortar and pestle. The homogenate was subsequently moved to a centrifuge tube and spun for 10 min at room temperature at 3000 × g. Following collection, 10 mL of distilled water were added to the supernatant. Subsequently, 10 mL of distilled water were used to dilute 1 mL of the supernatant for estimating purposes. The standard solution was first pipetted into a series of test tubes designated S1, S2, S3, S4, and S5, in the following amounts: 0.2, 0.4, 0.6, 0.8, and 1‐mL aliquots. Subsequently, 0.5 and 1 mL of the sample's protein extract were transferred into two additional test tubes labeled T1 and T2, respectively. One milliliter of distilled water was the only substance present in each test tube, and this same milliliter of water was used as the blank. Next, each test tube, including the blank, received 5 mL of the alkaline copper solution (0.5% copper sulfate in 1% potassium sodium tartrate + % sodium carbonate in 0.1 N sodium hydroxide). After thoroughly vortexing each test tube, they were left to stand at room temperature for 10 min. After that, each test tube, including the blank, received 0.5 mL of Folin–Ciocalteu reagent (1 N). After being vortexed, the test tubes were allowed to sit at room temperature for half an hour in the dark. Using a spectrophotometer, the absorbance of the blue color that had developed was calculated at 660 nm in relation to the reagent blank. Plotting the BSA concentration on the x‐axis and the corresponding absorbance on the y‐axis resulted in the creation of a standard graph. Ultimately, the quantity of total proteins in the sample was determined and reported as g BSA equivalents/100 g of sample.

#### 2.5.6 Estimation of Carbohydrate Content in PhPC

The following formula was used to calculate the sample's percentage carbohydrate content by subtracting crude fiber from a mathematical difference:

$$\begin{array}{c} \text{Carbohydrate}\;\left(\%\right)=\\ 100–\left(\text{moisture}+\text{crude protein}+\text{crude}\;\mathrm{fat}+\mathrm{ash}\right) \end{array}$$

#### 2.5.7 Quantification of Minerals and Heavy Metals in PhPC

The analysis of Minerals and heavy metals of PhPC was determined at the Chemistry Division, Atomic Energy Centre, Dhaka (AECD), in collaboration with the Department of Biochemistry and Molecular Biology, University of Chittagong, by using Atomic Absorption Spectrophotometer (AAS) – (Flame and Flameless).

### 2.6 Assaying the Protein Denaturation Inhibitory Effect of PhPC

#### 2.6.1 Inhibition of Bovine Serum Albumin Denaturation Test

With a minor modification, Williams et al.'s (2002) methodology was used to assess the in vitro anti‐inflammatory test for the suppression of bovine serum albumin protein denaturation. Various concentrations (800–100 μg/mL) of diclofenac sodium (DS) and PhPC (Protein hydrolysate of *P. conica* ) were prepared in distilled water to make a standard DS solution and PhPC solution. Eight (8.0) mg of a sample or standard was dissolved in 10 mL of distilled water to make a stock solution of a concentration of 800 μg/mL. The resulting stock solution was diluted with distilled water to give concentrations of 400–100 μg/mL. Glacial acetic acid was used to raise the pH of the 0.05 M tris‐buffer solution to 6.5 in order to create the stock solution of 0.8%, w/v BSA. A 2.5 mL of BSA solution was mixed with 0.5 mL of different concentrations of the sample or standard drug (Diclofenac sodium). Distilled water served as a negative control. Then the mixture of all test tubes was incubated at 37°C for 30 min. After cooling down, the solutions were heated for 15 min at 67°C in a water bath. Three duplicates of the 4.0 mL cuvettes' turbidity were then measured at 660 nm using a spectrophotometer. Each compound's degree of inhibition of denaturation or precipitation of the BSA from the solution was determined using the formula below:

$$\begin{array}{c} \%\text{of anti}-\text{denaturation activity}=\\ \left[\left({\text{Absorbance}}_{\text{control}}-{\text{Absorbance}}_{\text{treatment}}\right)/{\text{Absorbance}}_{\text{control}}\right]\\ \times 100\%\text{of anti}-\text{denaturation activity}\\ =\%\text{of protein denaturation inhibition}\\ =\%\text{of anti}-\text{inflammation} \end{array}$$

#### 2.6.2 Inhibition of Egg Albumin Denaturation Test

The procedure was modified slightly to determine the in vitro anti‐inflammatory test for the denaturation of egg albumin protein (Chandra et al. n.d.). Various concentrations (3200–400 μg/mL) of diclofenac sodium and PhPC were prepared in distilled water to make a standard aqueous and sample solution. Thirty‐two mg of a sample or standard was dissolved in 10 mL of distilled water to make a stock solution of a concentration of 3200 μg/mL. This stock solution was diluted with distilled water to give concentrations of 1600, 800, and 400 μg/mL. A 1% stock egg albumin solution was prepared using phosphate‐buffered saline (pH 6.5). Reaction mixtures were prepared by mixing 2 mL of sample from each different concentration and 3 mL of egg albumin solution. A similar procedure was used for the reference drug diclofenac sodium. Apart from this, distilled water was used as a negative control. The reaction mixtures were kept at 37°C ± 2°C for 30 min while they were incubated in a water bath. Subsequently, the mixture was heated to 60°C for 10 min and allowed to remain there for 5 min. The reaction mixture was then given 10 min to cool down at room temperature. A spectrophotometer was used to measure the absorbance of the reaction mixture at 660 nm for each concentration both before and after denaturation. After three repetitions of each test, the mean absorbance was noted. The percentage of inhibition of protein was determined on a percentage basis to control using the following formula.

$$\begin{array}{c} \text{Percentage inhibition}\;\left(\%\right)\\ =\frac{\text{Absorbance of control}‐\text{Absorbance of sample}}{\text{Absorbance of control}}\times 100 \end{array}$$

#### 2.6.3 Membrane Stabilizing Assay

The in vitro anti‐inflammatory activity of the membrane stabilization test was determined by a method described by (Shinde et al. 1999) with slight modification. Various concentrations (1600–200 μg/mL) of the reference drug diclofenac sodium and PhPC sample were prepared in distilled water to make a sample solution. Using 10 mL of distilled water and 16 mg of a sample or standard, a stock solution with a concentration of 1600 μg/mL was created. The stock solution was further diluted with water to make concentrations of 800–200 μg/mL. Wistar albino rats were used to provide the blood, which was then combined with an equivalent volume of sterilized Alsever solution (2% dextrose, 0.8% sodium citrate, 0.05% citric acid, and 0.42% sodium chloride in water) in accordance with an animal ethics protocol approved by the University of Chittagong. After centrifuging the blood for 10 min at 3000 rpm, the packed cells were cleaned with isosaline (0.85%, pH 7.2). With isosaline, a 10% (v/v) RBC suspension was created. In this experiment, two different kinds of media—distilled water and hypo saline—were utilized. 1 mL of phosphate buffer (0.15 M, pH 7.4), 2 mL of hyposaline, 0.5 mL of RBC suspension, and 1 mL of diclofenac sodium made up the test mixture in a hyposaline medium. Similarly, 1 mL of PhPC was utilized to create various sample concentrations. Two milliliters of distilled water were utilized as the control in the distilled water medium rather than hyposaline. After 30 min of incubation at 37°C, each assay mixture was centrifuged once more. A spectrophotometer set at 560 nm was used to determine the amount of hemoglobin present in the supernatant solution. By assuming that 100% of the hemolysis would occur in the presence of distilled water, the percentage of hemolysis was computed.

The following formula was used to determine the proportion of stability or protection of the RBC membrane. For every category, there were three replications kept.

$$\begin{array}{c} \%\text{of protection}\\ =100–\left({\text{Absorbance}}_{\text{sample}}÷{\text{Absorbance}}_{\text{control}}\right)\times 100 \end{array}$$

#### 2.6.4 Anti‐Inflammatory Action Against RAW264.7 Macrophages Activated by LPS

##### 2.6.4.1 Cell Lines, Culture Condition, and Morphological Study Using an Inverted Light Microscope

The American Type Culture Collection (ATCC) provided RAW264.7 macrophages, which were cultivated in Dulbecco's modified Eagle's medium (DMEM) supplemented with 10% (v/v) fetal bovine serum (FBS) and 1% (v/v) P/S at 37°C in a humidified environment with 5% CO_2_. As stated in a published publication, the morphological study was carried out (Tor et al. 2015). In short, a 6‐well plate was seeded with 3 × 105 cells per well, and the cells were incubated for 24 h to allow adhesion. Following the removal of the medium, treated and untreated cells were once more cultured for 72 h in a fresh medium that contained the treated samples in their IC_50_ concentration. After incubation, an inverted light microscope (Olympus, Tokyo, Japan) was used to observe morphological changes in the cells.

##### 2.6.4.2 RT‐qPCR for Analysis of Gene Expression

The total RNA was isolated from the control (DMSO only) and treated RAW 264.7 macrophages (stimulated by 1 μg of LPS) using TRIzol reagent (Invitrogen), and a NanoDrop spectrophotometer was used to quantify the extracted RNA. About 1 μg of purified total RNA was then reverse‐transcribed into cDNA in a total volume of 10 μL using the High‐capacity RNA to cDNA Master Mix kit (Applied Biosystems) according to the manufacturer's guidelines. The newly transcribed cDNA was used as a template, and RT‐qPCR was performed using SYBR qPCR mix (Nippon Gene) following a published protocol (Haque et al. 2020). All the primers used for quantifying the inflammatory‐related genes are shown in the following Table 1. The ΔCt values were calculated, and the standard deviation of the Ct values of the ΔCt values was also calculated, which were used to determine the ΔΔCt values. And then, all data were collected and calculated as 2^−△△Ct^.

**TABLE 1:** The sequences and base pairs of forward and reverse primers.

TNF‐α	Forward: ACG GCA TGG ATC TCA AAG AC
	Reverse: GGT CAC TGT CCC AGC TT
iNOS	Forward: GTG GTG ACA AGC ACA TTT GG
	Reverse: GGC TGG ACT TTT CAC TCT GC
IL‐1β	Forward: GAG TGT GGA TCC CAA GCA AT
	Reverse: CTC AGT GCA GGC TAT GAC CA
IL‐6	Forward: AGT TGC CTT CTT GGG ACT GA
	Reverse: GCC ACT CCT TCT GTG ACT CC
COX‐2	Forward: TCC TCC TGG AAC ATG GAC TC
	Reverse: TGA TGG TGG CTG TTT TGG TA
GAPDH (housekeeping gene)	Forward: TGC TCG AGA TGT CAT GAA GG
	Reverse: TTG CGC TCA TCG TAG GCT T

#### 2.6.5 Nitric Oxide Radical Scavenging Activity

With a small modification, the technique outlined by Sreejayan and Rao (1997) was used to determine nitric oxide radical scavenging. In this experiment, 150 min were spent at room temperature mixing various concentrations of 100 μL PhPC extracts dissolved in water with 1.5 mL of sodium nitroprusside (10 mM) in phosphate‐buffered saline (pH 7.4). The control was the same reaction mixture without the extract but with the same volume of water. 1.5 mL of Griess reagent (1% sulfanilamide, 2% H_3_PO_4_, and 0.1% *N*‐(1‐naphthyl) ethylenediamine dihydrochloride) were added following the incubation period. At 546 nm, the chromophore's absorbance was measured in relation to the blank. A positive control was ascorbic acid.

Ascorbic acid was used in a duplicate run of the experiment to assess the activity. One can compute nitric oxide radical scavenging using this formula:

Scavenging activity of Nitric oxide radical (%) = [(Abs_Conrol_ − Abs_sample_)/Abs_Conrol_] × 100.

Abs stands for Absorbance. The % of scavenging activity was set against concentration to calculate IC_50_ (Inhibition concentration 50) using linear regression analysis.

#### 2.6.6 Assaying the Anti‐Hemolytic Effect of PhPC

The antihemolytic activity of PhPC was assessed by a method as described by Alinezhad et al. (2013) with slight modification. In this experiment, H_2_O_2_ is added to rat erythrocytes to cause hydroxyl radical damage. This results in the RBC rupturing and its contents leaking out, giving the medium the red color of hemoglobin. Various concentrations (1600–200 μg/mL) of ascorbic acid (standard) and PhPC were prepared in distilled water to make a sample solution. A stock solution with a concentration of 1600 g/mL was created by dissolving 16.0 mg of a sample or standard in 10 mL of distilled water. Water was used to dilute the stock solution to give concentrations of 800–00 μg/mL. Wistar albino rats were used to donate blood, and the blood was combined with the same volume of sterilized Alsever solution. After centrifuging the blood for 10 min at 3000 rpm, the packed cells were cleaned with isosaline (0.9%) until a clear solution was achieved. With isosaline, a 10% (v/v) RBC suspension was created. After that, 0.5 mL of 10% erythrocyte suspension was mixed with 1 mL of various sample or standard concentrations, and 4 mL of phosphate‐buffered saline was added. Using distilled water as a negative control was used. After allowing the mixture to sit at room temperature for 5 min, 0.2 mL of H_2_O_2_ solution was added to cause the membrane lipids to oxidatively degrade. For 3 h, the reaction mixture was gently shaken while being incubated at 37°C. The reaction mixture was incubated, and after 10 min at 4°C and 3000 rpm centrifugation, the amount of hemolysis was measured by measuring the absorbance at 540 nm, which corresponds to hemoglobin liberation.

$$\%\text{of inhibition}=\left[\left({\mathrm{Abs}}_{\text{control}}-{\mathrm{Abs}}_{\text{sample}}\right)÷{\mathrm{Abs}}_{\text{control}}\right]\times 100$$

IC_50_ was calculated using the percentage inhibition.

#### 2.6.7 In Vitro Antibacterial Screening of Sample

Using Bauer's (1966) disk diffusion method, PhPC's antibacterial activity was assessed. The method's basic tenet is the direct connection between the size of the inhibition area of germ colonies formed around the tested drug and the degree of sensitivity. The procedure was modified by using sterile filter paper discs, the same size as those used in antibiograms, for the antibiotic discs. Purified colonies of every microorganism were used in the procedure, and they were suspended in sterile saline until the turbidity matched the McFarland number reached of 0.5 (1.5 × 108 CFU/mL with absorbance ranging from 0.08 to 0.13 at 625 nm). A loopful from each cultured organism was cotton‐swabbed onto Muller Hinton agar (HiMedia Laboratories, India). Sterile paper discs were impregnated with 5 μL of the protein hydrolysate sample (PhPC 100 μg/disc) and placed on the agar using sterile forceps. After that, the plates were placed upside down in a refrigerator set at 4°C for roughly an hour to give the substance enough time to diffuse. The plates were then inverted and incubated for 24 h at 37°C. The sample was examined in duplicate, and the zones of inhibition (including the 6 mm disk) were measured and recorded in millimeters to determine the antibacterial activity. To evaluate the antibacterial potency of the commercial antimicrobial agent with the protein hydrolysate, parallel analysis research was carried out using Ciprofloxacin (BBLTM Sensi‐DiscTM) at a concentration of 5 μg/disc as a standard.

##### 2.6.7.1 Minimum Inhibitory Concentration of PhPC

The minimum Inhibitory Concentration (MIC) of the protein hydrolysate was determined by the modified Wiegand method (Wiegand, Hilpert, and Hancock 2008) using a 96‐well microtiter plate. In each well of the microplate, 50 μL of the peptide at concentrations ranging from 40 mg/mL to 3.9 × 10^−2^ mg/mL (serial dilution) was placed, and 40 μL of nutrient broth and 10 μL of a bacterial suspension with an inoculum size of 1.5108 CFU/mL (McFarland No. 0.5) were introduced to make the total volume of each well 100 μL. Thereafter, the plates were covered and incubated at 37°C for 24 h. After incubation, 40 μL of 0.5 mg/mL 3‐(4, 5‐dimethylthiazol‐2‐yl)‐2,5‐diphenyltetrazolium bromide (MTT) was mixed in the wells and allowed to incubate for 30 min at 37°C. The peptide extract's minimum inhibitory concentration (MIC) was found to be the lowest amount that prevented the test organism's development. Regarding MTT, a well's light‐yellow color indicates that there hasn't been any microbial growth, whereas a well's purple color indicates that growth has occurred. All tests were performed in triplicate. Distilled water and ciprofloxacin were respectively used as a negative and positive control.

#### 2.6.8 Assaying of Thrombolytic Effects of PhPC

The clot lysis experiments were conducted as previously reported (Rahman, Sultana, and Emran 2013). In summary, eight distinct pre‐weighed sterile microcentrifuge tubes (0.5 mL/tube) were filled with 4 mL of venous blood extracted from the healthy volunteers, and the tubes were incubated at 37°C for 45 min. The serum was totally removed without affecting the clot after it had formed, and the weight of each tube containing a clot was once more measured to ascertain the clot weight (clot weight = weight of clot‐containing tube – weight of tube alone). Each microcentrifuge tube holding a pre‐weighed clot had a separate addition of 100 μL of PhPC. To the numbered control tubes, 100 μL of SK was added as a positive control, and 100 μL of distilled water was added as a negative non‐thrombolytic control. After 90 min of incubation at 37°C, the clot lysis of each tube was monitored. Following incubation, the fluid that had been discharged was collected, and tubes were weighed once more to see how the weight had changed following clot disruption. A percentage of clot lysis was calculated by comparing the weight acquired before and after clot lysis. The 20 individuals' blood samples were used to repeat the experiment. In order to prevent hormonal effects from influencing the assay results, female volunteers were excluded. The experiment was conducted following the institutional ethical guidelines of the Faculty of Biological Sciences, University of Chittagong [approval no. AERB_FBSCU_20230213 (1)].

##### 2.6.8.1 Statement on Informed Consent of the Donors

The donor consent form was produced in the light of the consent concept adopted by Rahman, Sultana, and Emran (2013). The volunteer donors were supplied a consent form that informed them of the details of the research project, including stepwise approaches, inclusion and exclusion criteria of the donors, the amount of blood to be taken, and possible discomfort of blood collection.

### 2.7 Statistical Analysis

The data is shown as Mean ± SEM. Using the Statistical Package for Social Science (SPSS, Version 21.0, IBM Corporation, NY) statistical software, one‐way analysis of variance was used to evaluate the data. For multiple comparisons, Tukey's Post Hoc test was then performed. At *p* < 0.05, values were deemed significant.

## 3 Results

The concentration of protein hydrolysate of *P. conica* (PhPC) in the supernatant was found to be 381.125 mg/g weight of lyophilized preparation, which is equivalent to BSA (standard solution) based on the curve and calculation.

### 3.1 Nutritional Status of PhPC

#### 3.1.1 Proximate Analysis, Heavy Metals, and Mineral Composition of PhPC

Proximate analysis was used to estimate the relative amounts of moisture, ash, protein, lipid, and carbohydrate in the PhPC sample (Table 2). The moisture content of PhPC was noticed to be 71.39% ± 0.12%. The ash, protein, lipid, and carbohydrate contents of PhPC are presented in Table 2.

**TABLE 2:** Proximate composition of PhPC freshwater mollusk (% dry matter basis).

SL no.	Amount (%)
Percentage (%) of moisture	71.39 ± 0.12
Percentage (%) ash	4.91 ± 0.22
Percentage (%) of crude fat	5.32 ± 0.00
Percentage (%) of total protein	15.55 ± 0.07
Percentage (%) of carbohydrate	2.83 ± 0.01

The contents of heavy metals and mineral composition of PhPC are summarized in Table 3. The amounts of Lead (Pb), Cadmium (Cd), Chromium (Cr), Arsenic (As) were found to be within the prescribed limit of toxicity and hazards. The amounts of Sodium (Na), Potassium (K), Iron (Fe), Calcium (Ca), Magnesium (Mg) of the sample are presented in Table 3. The sample PhPC was found to be enriched with the highest amount of Calcium 4693 ± 140 mg/kg.

**TABLE 3:** Minerals and heavy metal contents of the protein hydrilysate of *Paludomas conica*.

Name of the elements	Unit	Concentration
Lead (Pb)	ppm	< 0.1
Cadmium (Cd)	ppm	0.32 ± 0.02
Chromium (Cr)	ppm	0.56 ± 0.05
Arsenic (As)	ppm	0.56 ± 0.04
Sodium (Na)	mg/kg	332.22 ± 29.90
Potassium (K)	mg/kg	195.73 ± 17.61
Iron (Fe)	mg/kg	996.67 ± 59.80
Calcium (Ca)	mg/kg	4693 ± 140
Magnesium (Mg)	mg/kg	1185 ± 59

### 3.2 Anti‐Inflammatory Effect of PhPC

#### 3.2.1 Effect of PhPC on Bovine Serum Albumin and Egg Albumin Denaturation Inhibition as Well as Membrane Stabilization

**FIGURE 2:**
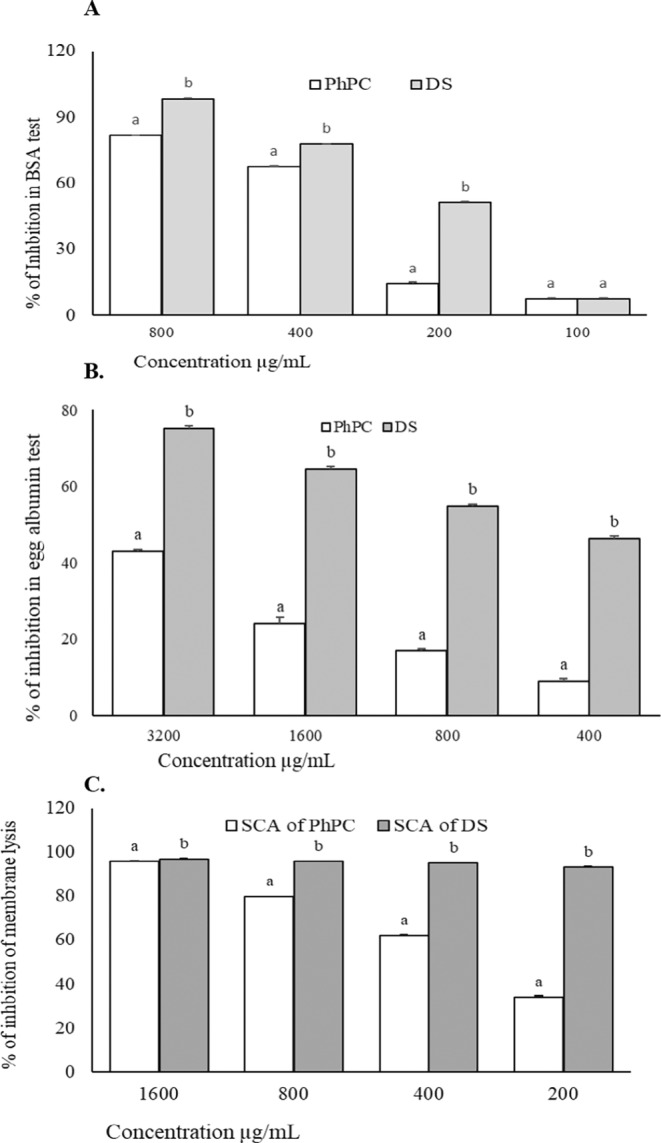
Comparative inhibition power of Diclofenac sodium and PhPC; (A) Inhibitory effect in BSA protein denaturation assay; (B) Inhibition assay by egg albumin test; (C) Membrane stabilizing effect. Data were presented as Mean ± SEM (*n* = 5).

The PhPC's bovine serum albumin denaturation inhibition activity increases with aggregate concentration in this experiment. The dose‐dependent denaturation inhibition of diclofenac sodium and PhPC was maximized at 800 μg/mL of DS and PhPC. The IC_50_ value, calculated by linear regression analysis from the graph of inhibition activity versus concentration, of PhPC was found to be 431.39 μg/mL, which was statistically significant (*p* < 0.05) compared to that (155.89 μg/mL) of diclofenac sodium. The IC_50_ values of PhPC and diclofenac sodium are summarized in Table 4, and their comparative scavenging effects are shown in Figure 2A.

**TABLE 4:** Anti‐inflammatory effects of Protein hydrolysate of *Paludomas conica* (PhPC).

Anti‐inflammatory models	IC_50_ value (μg/mL)	
	Standard (DS)	Sample (PhPC)
BSA protein denaturation assay	155.89	431.39
Egg albumin test	458.87	3788.72
Membrane‐stabilizing assay	17.33	285.25
Nitric oxide scavenging effect	16.67	1164.91
Antihemolytic effect	503.24	1907.68

Abbreviations: DS, diclofenac sodium; PhPC, *Paludomas conica*.

Like the standard, an increase in PhPC concentration enhances the egg albumin denaturation inhibition activity in this experiment. Diclofenac sodium and PhPC inhibited the denaturation in a dose‐dependent approach. Maximum inhibition of standard and PhPC was achieved at their concentration of 3200 μg/mL. Diclofenac sodium and PhPC were found to show inhibition concentration (IC_50_) values of 458.87 and 3788.72 μg/mL, respectively. Table 4 showed the IC_50_ values, and Figure 2B showed the comparative scavenging effects.

The increased concentration of PhPC maximized the RBC membrane lysis inhibition. Diclofenac sodium and PhPC inhibited the membrane lysis in a dose‐dependent manner. The highest scavenging effects of both standard and PhPC were found to be achieved by 1600 μg/mL. The IC_50_ value of PhPC was found to be 285.25 μg/mL, which was statistically significant compared to that of diclofenac sodium, the reference membrane‐stabilizing agent. The data of the comparative scavenging effect and IC_50_ values of DS and PhPC are shown in Figure 2C and Table 4.

#### 3.2.2 Effect of PhPC on the Morphology of LPS‐Stimulated RAW264.7 Macrophages

**FIGURE 3:**
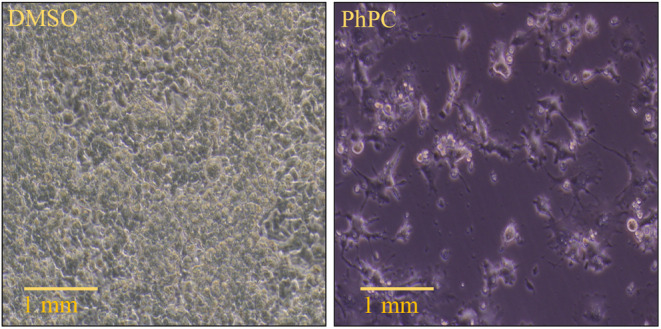
Effect of PhPC on cellular morphology. The number of treated cells was significantly reduced at 72 h posttreatment when compared with the untreated cells. Cell detachment, cell rounding, cytoplasmic condensation, and cell shrinkage are the symptoms of cell death induced by tested sample treatment. The figure shows the comparative morphological changes in the DMSO‐treated and PhPC‐treated cells.

The characteristics of cell death in treated samples include changes in cellular morphology. When compared to the untreated cells, the number of treated cells was considerably lower 72 h after treatment. The symptoms of cell death brought on by the treatment of examined samples include cell separation, rounding, cytoplasmic condensation, and shrinkage (Figure 3).

#### 3.2.3 Effect of PhPC on the Expression of COX‐2, iNOS, Il‐1β, Il‐6, and TNF‐α mRNA Levels in LPS‐Induced Macrophage Cells (RAW 264.7)

**FIGURE 4:**
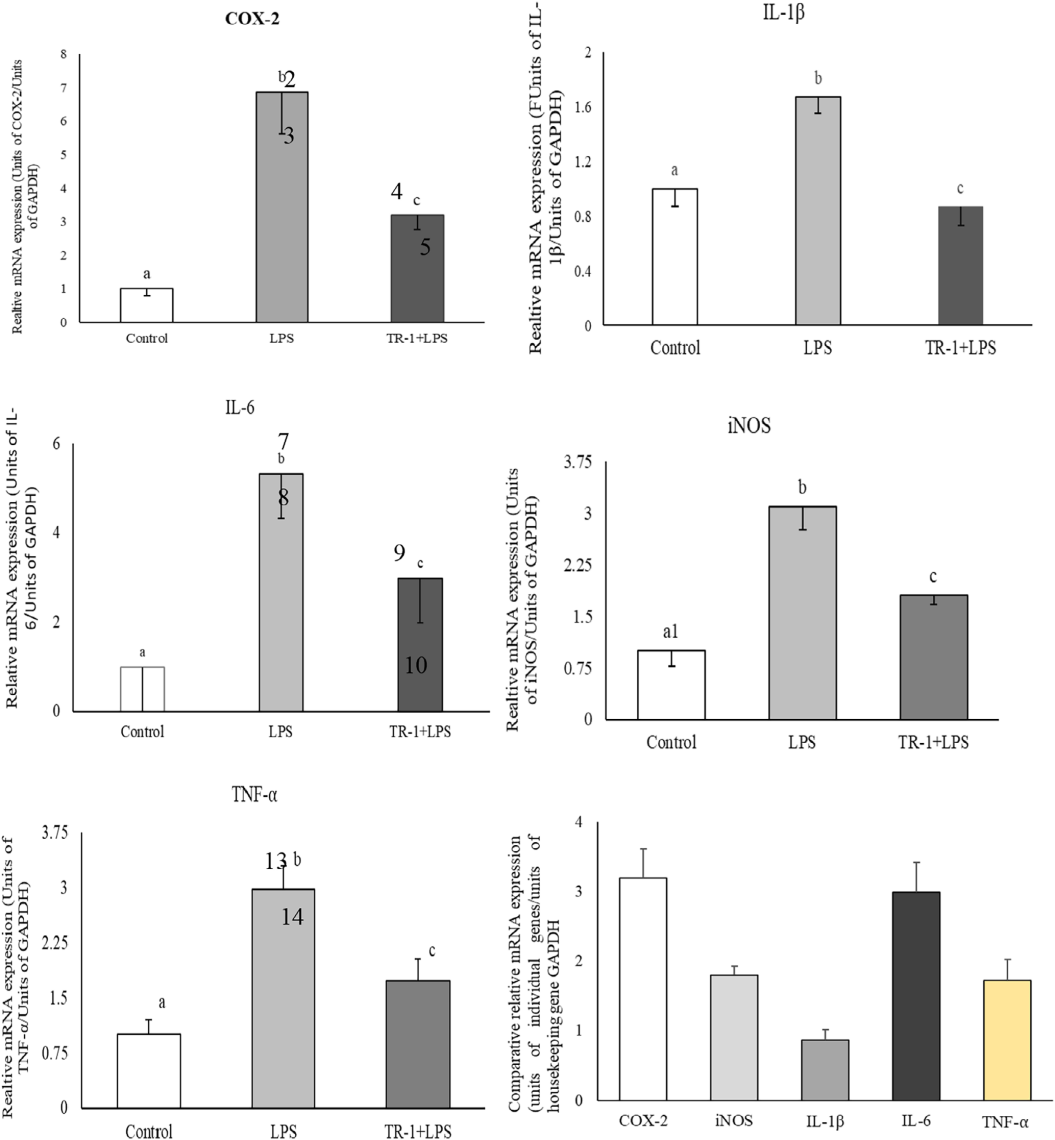
Comparative mRNA expression of the COX‐2, iNOS, Il‐1β, Il‐6, and TNF‐α following treating the RAW 264.7 cells with either LPS or a combination of LPS and tested sample Ph. Data were presented as Mean ± SEM (experiments were done in triplicate).

The effect of PhPC on LPS‐stimulated inflammatory enzymes and proinflammatory cytokines in terms of the mRNA expression levels of COX‐2, iNOS, Il‐1β, Il‐6, and TNF‐α in RAW 264.7 cells is presented in Figure 4. The results showed that LPS stimulation induced the mRNA expression of iNOS, COX‐2, Il‐6, Il‐1β, and TNF‐α compared to the unstimulated cells, and these up‐regulated genes were inhibited by the test sample PhPC. Though the tested sample does not have a similar suppressive role to all the tested genes. However, it was revealed that the corresponding genes INOS, COX‐2, IL‐1, IL‐6, and TNF‐α had their mean values of 6.853, 3.091, 1.672, 5.329, and 2.981 of mRNA expression for LPS‐induced cells downregulated to 3.192, 1.803, 0.871, 2.989, and 1.732 folds in the PhPC‐treated cells.

#### 3.2.4 Effect of PhPC on Inhibition of Nitric Oxide Radicals

**FIGURE 5:**
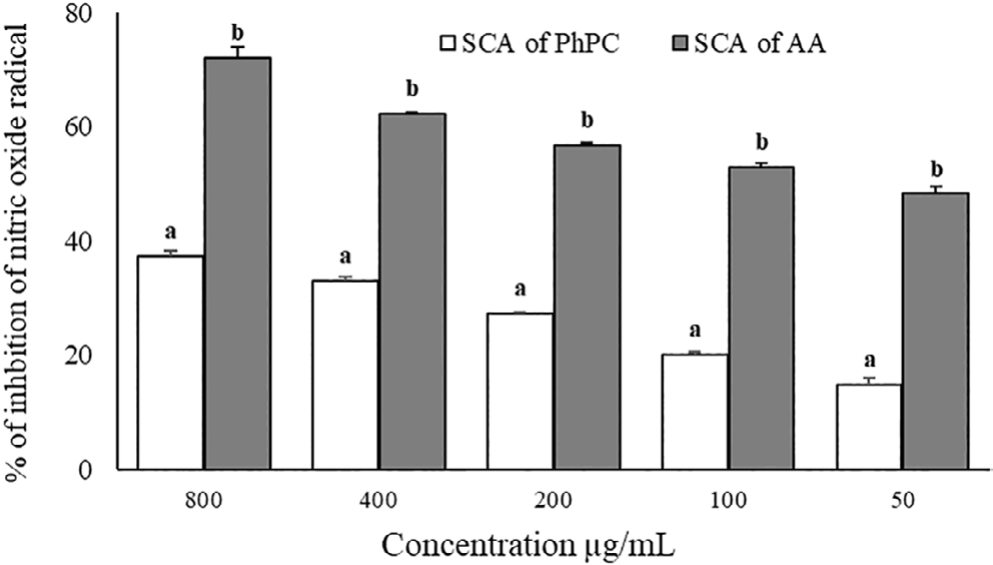
Comparative nitric oxide inhibition activity of ascorbic acid and *PhPC*; Data are presented as Mean ± SEM. Data are analyzed by one‐way ANOVA using SPSS (version 21.0, IBM, NY) followed by Tukey's post hoc test for multiple analyses. Values are considered significant at *p* < 0.05. The superscript letters (a, b) indicate the level of significance among the treatment groups.

Figure 5 displays the data on the comparative scavenging effect, and Table 4 provides a summary of the IC50 values. The results displayed an increased nitric oxide scavenging effect of *PhPC* with the increase in concentration. In a dose‐dependent inhibition assay, the maximum scavenging of both ascorbic acid (reference scavenging agent) and PhPC was gained at the concentration of 800 μg/mL.

### 3.3 Effect of PhPC on Hemolysis Inhibition

**FIGURE 6:**
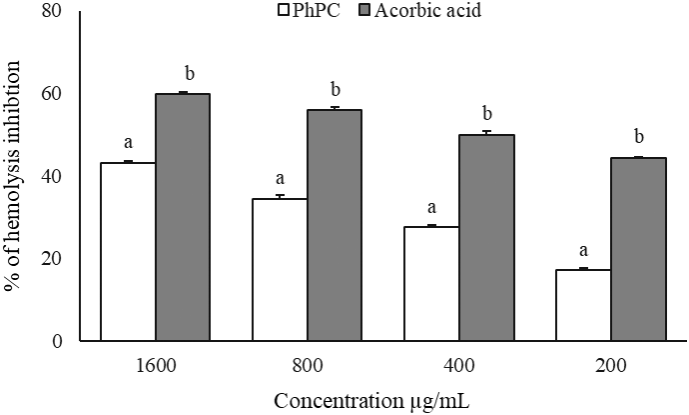
Relative hemolysis inhibition power of and PhPC and ascorbic acid. Data are presented as Mean ± SEM. Data are analyzed by one‐way ANOVA using SPSS (version 21.0, IBM, NY) followed by Tukey's post hoc test for multiple analyses. Values are considered significant at *p* < 0.05. The superscript letters (a, b) indicate the level of significance among the treatment groups.

The red blood cell (RBC) membrane damage inhibition of PhPC and ascorbic acid (a reference agent) was increased with concentration (Figure 6). They were found to show the highest effect at the concentration of 1600 μg/mL. The inhibition concentrations of PhPC and ascorbic acid were displayed to be 503.24 and 1907.68 μg/mL, while the values for PhPC were statistically (*p* < 0.05) insignificant compared to the reference standard.

### 3.4 Effect of PhPC on Microbial Inhibition

**FIGURE 7:**
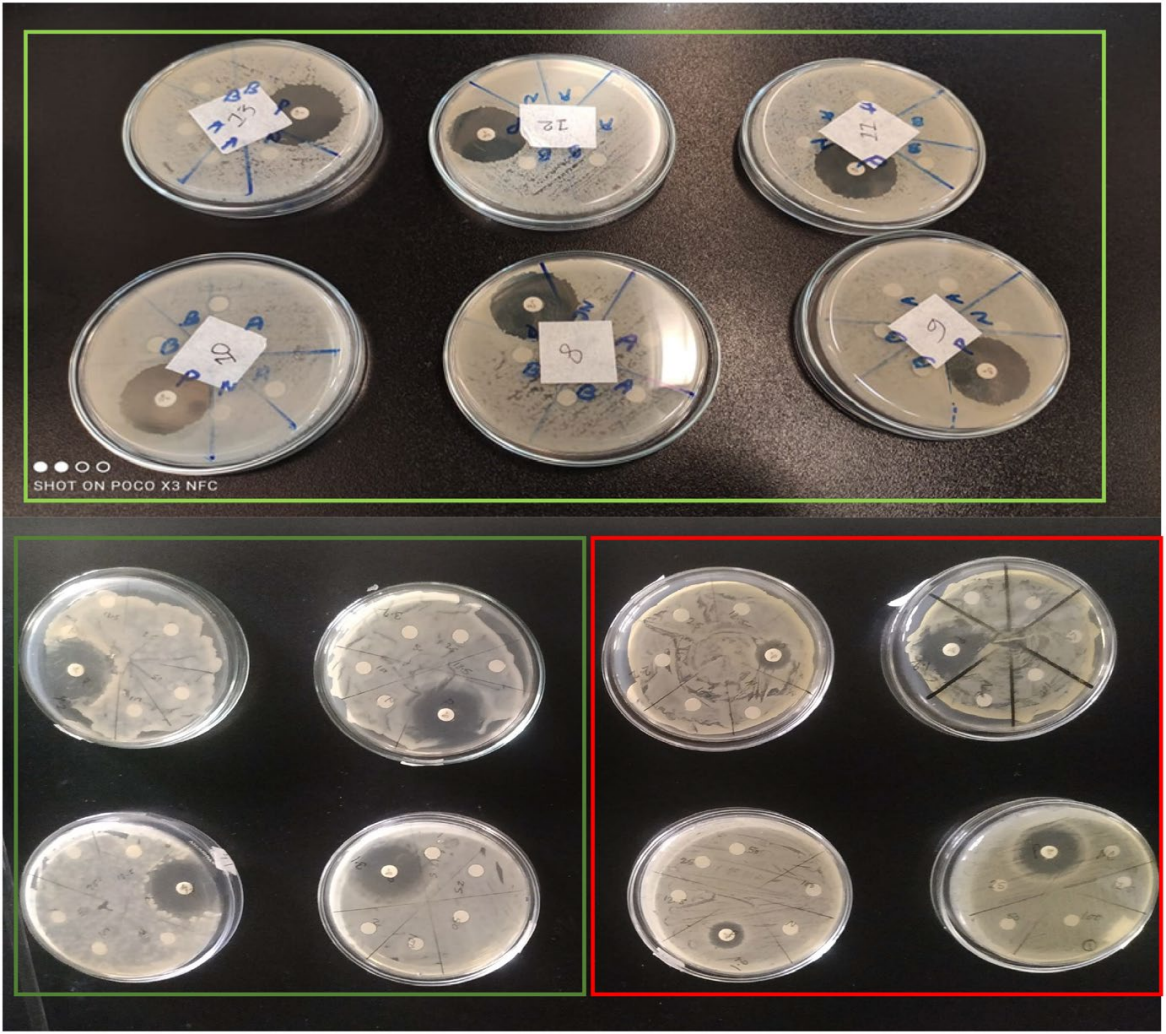
Antimicrobial effect of protein hydrolysate. Figure shows the zones of inhibition are shown in as *Shigella flexneri* (10.5 ± 0.57), Enterobacter cloaceae (9.5 ± 0.57), *Pseudomonas aeruginosa* (10.0 ± 0.0), *Shigella dysenteriae* (9.5 ± 0.57), and *Salmonella paratyphi* (8.5 ± 0.57). Antibacterial effects of ciprofloxacin and PhPC are marked by Green and Red boxes, respectively.

The antimicrobial activity of PhPC on 3 g‐positive and 8 g‐negative bacteria in the disc diffusion method is illustrated in Table 5, and the minimum inhibitory concentration (MIC) was presented in Table 6. The PhPC was found to be active against 5 g‐negative bacteria, while the highest growth inhibition was achieved against *Shigella flexneri,* 10.5 mm (Figure 7). The zone of inhibition of the standard antibiotic ciprofloxacin against the same bacterial strain was noticed as 34.5 mm. Both *Shigella dysenteriae* and *Pseudomonas aeruginosa* had a substantial MIC of 20 μg/mL, while *Shigella flexneri* and *Salmonella paratyphi* had an MIC of 10 μg/mL.

**TABLE 5:** Antibacterial effect of PhPC (zone of inhibition, mm).

Type	Test organism	Zone of inhibition	
		PhPC (100 μg)	Ciprofloxacin
Gram (+ve)	*Staphylococcus aureus* (ATCC 6538)	—	23.5 ± 0.57
	*Bacillus subtilis* (subcultured)	—	35.5 ± 0.57
	*bacillus cereus* (subcultured)	—	28.5 ± 0.57
Gram (−ve)	*E. coli* (ATCC 25922)	—	10.5 ± 0.57
	*Shigella flexneri* (ATCC 12022)	10.5 ± 0.57	34.5 ± 0.57
	*Enterobacter cloaceae* (ATCC 13047)	9.5 ± 0.57	26.5 ± 0.57
	*Pseudomonas aeruginosa* (subcultured)	10.0 ± 0.0	29.5 ± 0.57
	*Shigella dysenteriae* (subcultured)	9.5 ± 0.57	28.5 ± 0.57
	*Vibrio cholerae* (subcultured)	—	28.5 ± 0.57
	*Salmonella typhi* (subcultured)	—	30.5 ± 0.57
	*salmonella paratyphi* (subcultured)	8.5 ± 0.57	34.5 ± 0.57

*Note:* (−)‐indicates the insensitivity of the antibiotic against bacterial strains.

**TABLE 6:** Minimum inhibitory concentration of *PhPC* on various test organisms.

*Bacteria*	Minimum inhibitory concentration (Concentration μg/mL)											
	40.0	20.0	10.0	5.0	2.5	1.25	0.625	0.3125	0.156	0.078	0.039	NC
*Shigella flexneri*	−	−	**	+	+	+	+	+	+	+	+	+
*Enterobacter cloacae*	−	−	−	**	+	+	+	+	+	+	+	+
*Pseudomonas aeruginosa*	−	**	+	+	+	+	+	+	+	+	+	+
*Shigella dysenteriae*	−	**	+	+	+	+	+	+	+	+	+	+
*Salmonella paratyphi*	−	−	**	+	+	+	+	+	+	+	+	+

Abbreviations: **, minimum inhibitory concentration; +, growth; −, no growth.

### 3.5 Effect of PhPC on Thrombolytic Activity

**FIGURE 8:**
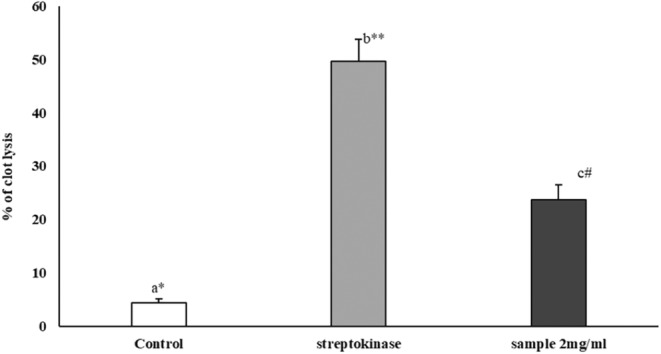
Thrombolytic effects of PhPC. Data are presented as Mean ± SEM (*n* = 20). Data are analyzed by one‐way ANOVA using SPSS (version 21.0, IBM, NY) followed by Tukey's post hoc test for multiple analyses. Values are considered significant at *p* < 0.05. The superscript letters (a–c) indicate the level of significance among the treatment groups. The mean clot lysis percentage difference was significant (*p*‐value < 0.001).

The effect of PhPC was observed through the clot lysis after 90 min of incubation of clotted blood with 100 μL of PhPC solution (test), streptokinase solution (positive control), and distilled water (negative control) at 37°C (Figure 8). Maximum clot lysis (49.70% ± 4.11%) was achieved by the reference drug streptokinase. The PhPC was found to show the clot lysis 23.72% ± 2.71%, which was statistically significant (*p* < 0.001) compared to the clot lysis (4.41% ± 0.73%) displayed by the negative control.

## 4 Discussion

Hemolytic diseases, associated with thrombosis, inflammation, and immune dysregulation, lead to the release of damage‐associated molecular patterns, including ADP, hemoglobin, and heme, which act through multiple receptors and signaling pathways, fostering a hyperinflammatory state. Hemolysis is often considered to be a logical explanation for the increased rate of bacterial infections. Therefore, the correlation between hemolysis and bacterial infections leading to inflammation is a discourse for a complete therapeutic regimen. This research evaluated the potential of PhPC, a mollusk‐based protein hydrolysate, in the inhibition of hemolysis and bacterial infection and inflammation.

Since ancient times, various peoples and cultures have used the therapeutic benefits of biodiversity. Along with the widely used plants in different countries of the world, animals are also used as traditional zootherapies in some cases (Lev 2003). The majority of people in developing countries have easy access and affordability to traditional animal therapies (Jain and Srivastava 2005). This research has used the protamex‐digested protein hydrolysate of a freshwater snail, *P. conica,* mostly available in the Hilly canals, to evaluate its biological actions, especially focusing on the anti‐inflammatory effects.

Although proximate analysis is one of the key commercial concerns, as food‐manufacturing companies need to ensure that their products comply with the appropriate laws and legal declaration, this is also significant at the consumer's level to ensure their nutritional adequacy from a particular source of food. All Bangladeshi freshwater snails studied so far have less than 50% of protein, while the protein, ash, fat, moisture, and carbohydrates vary based on different locations. The overall proximate analysis agrees with the published report on freshwater snails (Moniruzzaman et al. 2021). Monchanin et al. (2022) reported the permissible limit of heavy metals in foods or food products as Pb (0.01–3.00), Cd (0.05–2), As (0.1–0.2), and Cr (1.30) ppm (Monchanin et al. 2022). These limits of heavy metals confirmed that the protein hydrolysate of the studied *Paludomas* species could not be hazardous in terms of toxicity (Venugopal and Gopakumar 2017). Minerals added significant elements of enzymes, hormones, and other biological activators in human nutrition. Sodium, potassium, magnesium, calcium, iron, and phosphorus play a promising role in human nutrition (Arango Duque and Descoteaux 2014; U.S. Department of Agriculture, Aquiculture 2003), and their insufficiency can cause structural, biochemical, and pathophysiological changes (World Health Organization 2013). Our results showed that an ample amount of Na, K, Fe, Ca, and Mg (> 100 mg/kg) in the PhPC indicated its biological competency to support the biochemical and structural objects of humans through its consumption (Tabakaeva, Tabakaev, and Piekoszewski 2018).

The immune system's response to harmful stimuli, such as tissue damage, infection, or poisonous substances, is inflammation. Its goal is to get rid of irritants or pathogenic germs while promoting tissue repair. Chronic inflammatory diseases are typically treated with anti‐inflammatory drugs, but most of them are insufficient to control chronic responses and are also linked to negative side effects. A range of chronic inflammatory illnesses can be brought on by unchecked, protracted acute inflammation that progressively develops into chronic inflammation. As a result, numerous efforts are being made to create alternative and more focused anti‐inflammatory medicines using natural materials. Obtaining bioactive peptides with anti‐inflammatory properties from sustainable protein sources, such as edible invertebrates or fishery byproducts, is intensely important.

Denaturation of tissue proteins is one of the known causes of inflammatory and arthritic illnesses. The in vitro protein denaturation may be the cause of the production of autoantigens in some arthritic diseases. The increases in the absorbance of the protein hydrolysate and reference drug relative to the control suggested that the albumin protein had stabilized. Since the erythrocyte membrane and lysosomal membrane are similar, the HRBC method was selected for the in vitro assessment of the anti‐inflammatory characteristics. This is because the stabilization of the erythrocyte membrane suggests that the extract may also stabilize lysosomal membranes. Lysosomal membrane stability plays a critical role in regulating the inflammatory response by inhibiting the release of bactericidal enzymes and proteases, which are lysosomal components of activated neutrophils that induce further tissue inflammation and damage upon extracellular release (Rivera‐Jiménez et al. 2022; Wang et al. 2021). The significant inhibition of membrane stabilization and protein denaturation with the promising inhibition concentration (IC_50_ value less than the cut‐off value of 100 μg/mL) is in line with the anti‐inflammatory evidence for natural therapeutics.

Another method for predicting the effect is cellular morphology, which suggests that shrinkage of the cells because of osmotic loss of intracellular electrolyte and fluid components may be the cause of hypotonicity‐induced hemolysis. PhPC, or protein hydrolysate, has the potential to block certain processes that could otherwise promote or intensify the outflow of these intracellular components (Tkaczewska 2020). When exposed to lipopolysaccharide (LPS), macrophages get activated and release a variety of pro‐inflammatory proteins and cytokines. Excessive release of these mediators can lead to significant tissue damage and pathological alterations (Cao et al. 2019). So, the cellular changes may be reflected by the effect of the PhPC treatment, which reduces the release of inflammatory markers from the macrophages. Even though synthetic pharmaceuticals have advanced greatly in the last several years, they are discovered to have a variety of negative consequences, while the natural source of mollusk protein hydrolysate may have a special place because it has no negative effects.

Moreover, aberrant activation of enzymes linked to inflammation, such as lipoxygenase (LOX), cyclooxygenase‐2 (COX‐2), phospholipase A2 (PLA2), and inducible nitric oxide synthase (iNOS), is crucial in the emergence of inflammatory illnesses (Wang et al. 2021). Tumor necrosis factors (TNFs), like tumor necrosis factor‐α (TNF‐α), and cytokines of the interleukin family (ILs), like IL‐6, IL‐1β, and IL‐10, are examples of inflammatory mediators. TNFs mediate inflammation by interacting with various cellular components or receptors (Chen et al. 2017). Increased inflammation is usually reflected through these mediators. Therefore, therapeutic intervention mostly targets the decrement of the expression of inflammation‐inductive enzymes and mediators. In this research, the protein hydrolysate of the invertebrate snail *P. conica* has reduced the mRNA expression of iNOX, COX‐2, IL‐β, TNF‐α, and IL‐6 ranging from 1.5 to 2 folds. However, COX‐2 was maximally downregulated. COX‐2 inhibitors block the NF‐B pathway to produce their pharmacological effects. Since COX‐2 is the enzyme that generates reactive oxygen species (ROS), inhibiting COX‐2 dramatically reduces the amount of ROS in the upstream mechanism and keeps NF‐B in an inactive state of bondage to P‐IκB in the downstream, preventing the production of pro‐inflammatory cytokines like NO, PGE2, IL‐6, and TNF‐α (Ju et al. 2022). This implies the agreement of the results with established mechanistic views, thus indicating the anti‐inflammatory potentials of PhPC (Chan‐Zapata et al. 2019).

The excessive synthesis of reactive species (such as ROS and RNS) also plays a significant role in the emergence of inflammation and oxidative stress. The protein hydrolysate in this investigation showed a reduction in the NO in vitro model that was concentration dependent. Additional research has shown that grains containing protein derivatives have a suppressive effect on the generation of ROS or RNS. The NO generation of activated RAW 264.7 macrophages was shown to be inhibited by plant‐based protein hydrolysate by Ndiaye et al. (Ndiaye et al. 2012). Udenigwe et al. (2009) assessed the low molecular weight peptide treatment's effect on RAW 264.7 macrophages' ability to produce NO and noted that NO generation was inhibited (Udenigwe et al. 2013). Other protein hydrolysates also displayed a similar pattern, which would help to understand the mechanism preventing RNS and ROS by inhibiting the generation of superoxide dismutase (SOD), superoxide radical (O2.–) or inducible nitric oxide synthase (iNOS) expression, among other relevant markers. In our previous study, we found that PhPC remarkably increased the gene expression of Papraoxonase‐1 (PON‐1), superoxide dismutase‐1 (SOD‐1), and Catalase‐1 (CAT‐1) (Rafi et al. 2023). PON‐1 is an antioxidant enzyme that attenuates the production of the pro‐inflammatory monocyte, and SOD‐1 suppresses pro‐inflammatory immune responses by destroying superoxide radicals (Marsillach et al. 2009; Hwang et al. 2020). Catalase is a paramount antioxidative enzyme that reduces inflammatory damage, maintaining redox balance within cells and tissues, preventing the excessive accumulation of reactive oxygen species (Anwar et al. 2024).

Some recent research around the world focused on the common utilization of mollusks, which have recently been identified as possible sources of antibacterial properties. However, very few studies have guided the exploration of the advanced therapeutic effects of mollusks although they are found to produce a great diversity of novel functions due to their bioactive compounds. Protein hydrolysate generally comprises a mix of short peptides to exert their biological activity, including antibacterial effects (Kuppusamy and Ulagesan 2016). The highest activity of PhPc was found against *Shigella flexneri* and *Pseudomonas aeruginosa* which is a remarkable episode because *S. flexneri* is a notorious gram‐negative bacterium of multidrug resistance while the drug resistance of *P. aeruginosa* is widely reported. The biological attributes of protein hydrolysate are greatly affected by the characteristics of their molecular structure, such as amino acid composition, sequence, chain length, and net charge (Ahmed and Hammami 2019; Zhang et al. 2023). The antibacterial effects of the PhPC may have been affected by these characteristics. Additionally, hydrophobicity is considered significant because it makes it easier for peptides to interact with the cytoplasmic membranes of bacteria. Growth inhibition of some bacterial strains, especially the gram‐negative bacteria with PhPC in our research, greatly complies with the previous findings showing the antibacterial effects of mollusks as a new source of anti‐infective drug discovery.

Thrombotic diseases are widely recognized as one of the primary causes of death. Urokinase (uPA), Streptokinase (SK), and recombinant tissue‐plasminogen activator (rt‐PA) are among the common thrombolytic drugs used today to treat thrombosis conditions (Prasad et al. 2007). Their downsides in clinical application, however, include their side effects, which frequently include reports of severe immunoreactions, hemorrhagic outcomes, allergic reactions, and anaphylaxis, as well as their higher costs and lower fibrin specificity. Recently, the use of traditional medicinal animal creatures such as leeches, snakes, earthworms, and snails as a type of vicarious therapy has drawn more attention (Liu et al. 2022; Vernooij et al. 2009). The protein hydrolysate from our freshwater snail has given 23% of thrombolytic effects. These moderate effects could be improved through the purification of protein products or short peptides from the studied species, as evidenced by a novel fibrinolytic protein, EPF3. which was extracted from *P. vulgaris* using column chromatography, and its whole sequence was determined by de novo sequencing (Wu et al. 2020). Comprehensively, the results demonstrate that the nutritional values of PhPC co‐act with its reported antioxidative potential, which may attenuate the inflammatory and proinflammatory indices, leading to the inhibition of hemolysis and bacterial infection. This will also imply its prospects to be a great source of food supplements in inflammatory cases.

## 5 Conclusion

The results of this study have revealed that the protein hydrolysate of *Paludomas conica* is a rich freshwater invertebrate source with high mineral content, and the prescribed limit of heavy metals made it an excellent and safe food source. It could be used as an anti‐inflammatory agent that is able to suppress the studied inflammatory genes. The effects have been supported by nitric oxide scavenging potential. The antibacterial effects against selective gram‐negative bacteria and thrombolytic effects could be additional reports in this research. Additionally, we presume the presence of a wide variety of bioactive and therapeutic peptides and proteins, which could be affirmed by the purification of protein hydrolysate and purified protein that need to be characterized and sequenced to ensure their use alternative therapeutics to treat a wide variety of diseases, including inflammation.

## Author Contributions

**Tanvir Ahmed Siddique:** data curation (equal), methodology (equal), resources (equal). **Md. Khalid Juhani Rafi:** formal analysis (equal), methodology (equal). **Sumaiya Akter:** methodology (equal), visualization (equal). **Farhana Yesmin Bithy:** methodology (equal), resources (equal), software (equal). **Rasheda Aktar:** formal analysis (equal), writing – review and editing (equal). **Md. Asif Nadim Khan:** data curation (equal), validation (equal). **Mumtahina Majid:** methodology (equal), validation (equal). **Farjana Sultana:** methodology (equal), visualization (equal). **Srabonti Saha:** resources (equal), supervision (equal). **A. M. Abu Ahmed:** methodology (equal), writing – review and editing (equal). **Md. Atiar Rahman:** conceptualization (equal), project administration (equal), supervision (equal), resources (equal), reviewing and editing (equal).

## Conflicts of Interest

The authors declare no conflicts of interest.

## Data Availability

Data will be provided upon request.
